# A model-based comparison of dose reduction strategies for fixed-dose dolutegravir-containing regimens

**DOI:** 10.1093/jac/dkaf445

**Published:** 2025-12-12

**Authors:** Laura Dickinson, Laura Else, Willem D F Venter, Marta Boffito, Rhonda Brand, Catriona Waitt, Andrew Hill, Saye Khoo

**Affiliations:** Centre for Experimental Therapeutics, University of Liverpool, Liverpool, UK; Centre for Experimental Therapeutics, University of Liverpool, Liverpool, UK; Wits Ezintsha, University of the Witwatersrand Faculty of Health Sciences, Johannesburg, South Africa; HIV Services, Chelsea & Westminster Hospital NHS Foundation Trust, London, UK; Division of Gastroenterology, Hepatology & Nutrition, Magee Women’s Research Institute and University of Pittsburgh School of Medicine, Pittsburgh, USA; Centre for Experimental Therapeutics, University of Liverpool, Liverpool, UK; Infectious Disease Institute, Makerere University, Kampala, Uganda; Centre for Experimental Therapeutics, University of Liverpool, Liverpool, UK; Centre for Experimental Therapeutics, University of Liverpool, Liverpool, UK; Department of Infectious Diseases, University Hospitals of Liverpool Group, Liverpool, UK

## Abstract

**Background:**

Countries faced with an impending shortage of antiretrovirals need to conserve supplies and maximize treatment effectiveness while limiting harms. Potential strategies to achieve this could include reduced doses of fixed-dose tenofovir disoproxil–lamivudine–dolutegravir (TLD).

**Methods:**

Nonlinear mixed effects was applied to dolutegravir concentration-time data from three healthy volunteer studies (two including dolutegravir ‘washout’ over 10 and 15 days post-cessation), adapting a previously published model. The model was used to simulate (*n* = 1000) pharmacokinetic profiles of standard dolutegravir (one pill TLD daily) and four dose reduction strategies: half a pill TLD daily; one pill TLD every other day; 4 days on, 3 days off (4:3) and 5 days on, 2 days off (5:2) therapy. Proportions above and time below the EC_90_ (320 ng/mL) were determined and compared.

**Results:**

Seventy-three volunteers (60% female, *n* = 825 concentrations) were included in the model. Marked differences between simulated dose reduction regimens were observed, with alternate day, 4:3 and 5:2 dosing, associated with a lower proportion of dolutegravir trough above the EC_90_ (41.6%–62.1%, 2.0%–5.2%, 14.1%–20.7%, respectively) across 50, 70 and 90 kg compared with daily dosing at full or half doses (88.7%–99.8%). Of the four dose reduction strategies, 4:3 dosing exhibited the longest predicted time below target before the next due dose (median ∼2 days).

**Conclusions:**

Safety and effectiveness of TLD dose reduction strategies can only be established in clinical studies and cannot be extrapolated from models. However, these models can inform regimen design to limit the window of opportunity for emergence of resistance, i.e. where viral replication occurs in the presence of appreciable drug concentrations.

## Introduction

This year is a turning point for HIV. Decades of international efforts to expand testing and widen access to antiretrovirals for treatment and prevention have been put at risk through reductions in foreign aid. The abruptness of these cuts is likely to lead to a scarcity of antiretrovirals in several countries in Africa, and some will probably run out of medicines in the coming months.^1^ Notably the suspension of the US President’s Emergency Plan for AIDS Relief (PEPFAR) support has caused the widest disruption, with UNAIDS estimating an additional 6.6 million new HIV infections could occur worldwide and 4.2 million AIDS-related deaths by 2029 if PEPFAR was permanently discontinued.^2^ A modelling study determined that unmitigated reductions in international funding from all major donor countries could detrimentally affect the progress of the HIV response, disproportionally affecting Sub-Saharan African countries and vulnerable populations.^3^ Faced with a likely rise in late-stage illness and death,^3^ and the possibility of spread of drug resistance, strategies are necessary to help manage antiviral supply to save as many lives as possible while limiting any adverse impact on the effectiveness of antiretroviral therapy (ART).

Tenofovir disoproxil–lamivudine–dolutegravir (TLD) is the most commonly prescribed first-line ART regimen across low-income countries in Sub-Saharan Africa and elsewhere. There has been speculation as to whether dose reduction of fixed-dose combination regimens could be used in a crisis response.^1^ Of seven published randomized trials, four included non-nucleoside reverse transcriptase inhibitors and only two [the ANRS 170 QUATUOR study (*n* = 636) and BREATHER-Plus (*n* = 470)] included dolutegravir-based regimens; both studied the use of intermittent dosing e.g. 4 days on, 3 days off therapy and 5 days on, 2 days off therapy, respectively. Virological failure rates (defined as viral load >50 copies) of intermittent therapy at 48 weeks did not differ from daily treatment for ANRS 170 QUATUOR, with no DTG-related failures at 96 weeks.^4^ However, the BREATHER-Plus trial recently reported 5 days TLD with weekends off was inferior to daily intake at 96 weeks.^5^ Other dose reduction strategies (e.g. alternate day dosing, or half-dosing daily) have not been studied.

Modern ART regimens are associated with very high potency and long elimination half-lives, to the extent that drug concentrations at the end of the dose interval are still many times above the putative target, and these high ‘inhibitory quotients’ (together with prolonged dissociation kinetics of second-generation integrase inhibitors^6^) provide a significant cushion for late dosing. Could reducing the dose, or prolonging the dose interval be used to conserve precious stocks of drug while alternative funding solutions are negotiated? Such a strategy is not risk-free: not all regimens (or components within a fixed-dose combination) are equally robust, and people living with HIV will probably differ in their ability to tolerate dose reduction. Although second-generation integrase inhibitors have some of the highest genetic barriers to resistance, the prevalence of resistance is increasing with the global deployment of TLD.^7^

Tenofovir and lamivudine are prodrugs, metabolized within cells to their active moieties, tenofovir-diphosphate and lamivudine-triphosphate, which have half-lives in white blood cells of 2–6 days^8^ and 27 hours, respectively.^8–10^ This intracellular persistence is considerably longer than their plasma parent compounds, conferring greater flexibility for less-than-complete adherence to daily therapy.^11^ A dose reduction of lamivudine from 300 to 150 mg reported prolonged washout of intracellular lamivudine-triphosphate although lower concentrations were attained.^12^ For TLD dose reduction strategies, changes in plasma concentrations of dolutegravir following reduced dosing are likely to be the most critical component for success. In this study, we used mathematical models to simulate the likely population exposures of dolutegravir when TLD is dosed daily: half a pill daily; one pill every other day; 4 days on therapy, 3 days off therapy (4:3) or 5 days on therapy, 2 days off therapy (5:2) across a range of body weights. Since these models require validation using drug concentration measurements beyond the next due dose, we used data from rich sampling of healthy volunteers who discontinued dolutegravir in pharmacokinetic ‘washout’ studies or as controls in drug interaction studies.

## Methods

### Analysis population

Dolutegravir plasma concentrations were pooled from three clinical healthy volunteer studies which have been described fully elsewhere along with complete inclusion/exclusion criteria.^13–15^ The studies recruited male and non-pregnant, non-breastfeeding female adults without any significant medical conditions or taking therapies likely to interact with study medications. All studies received approval from the relevant authorities and conformed with the Declaration of Helsinki and participants provided written informed consent. Brief study descriptions were as follows:

- The Pittsburgh study (NCT04302896)^13^ determined plasma and urine drug concentrations of dolutegravir (plus either emtricitabine/tenofovir alafenamide or lamivudine/tenofovir disoproxil fumarate) over 14 days following drug cessation once dosed to steady-state. Participants without HIV were recruited at the Magee-Women’s Hospital Clinical Translational Research Centre (MWH CTRC, Pittsburgh, PA, USA). Participants were randomized 1:1 to receive dolutegravir 50 mg once daily in combination with either 200 mg emtricitabine/25 mg tenofovir alafenamide or 300 mg lamivudine/300 mg tenofovir disoproxil fumarate. Dosing over 15 days was directly observed or recorded by participants; participants were instructed to eat normally while taking the medication. Serial sampling was performed at pre-dose on days 1, 2 and 8 and before the final dose (0 hour) on day 15 and 1, 4, 24, 48, 72, 96, 168 and 336 hours post-final dose.
- SSAT061 (NCT02219217)^14^ determined the pharmacokinetic profile of dolutegravir and elvitegravir/cobicistat over 10 days following drug cessation once dosed to steady-state in volunteers without HIV recruited at Chelsea & Westminster Hospital (London, UK). Individuals were administered dolutegravir 50 mg once daily for 10 days. On the day of pharmacokinetic sampling a standardized breakfast (626 kcal) was provided for drug intake; serial sampling was performed pre-dose (0 hour), 2, 4, 8, 12, 24, 36, 48, 60, 72, 96, 120, 144, 168, 192 and 216 hours following the last dose.
- DolACT (NCT02242799)^15^ evaluated the pharmacokinetic drug-drug interaction between dolutegravir and artemether-lumefantrine or artesunate-amodiaquine the Infectious Diseases Institute, Makerere University (Kampala, Uganda). Dolutegravir 50 mg once daily was administered for 6 days with and without artemether-lumefantrine in a random sequence two-way crossover study or for 7 days with and without artesunate-amodiaquine in a parallel study. Study medication was taken with a standardized moderate fat breakfast and samples drawn at pre-dose (0 hour), 1, 2, 3, 4, 8, 12 and 24 hours post-dose.

Whole blood samples were collected from study participants in either K_2_EDTA^13,15^ or lithium heparin tubes.^14^ Plasma was separated by centrifugation and stored between −20 and −80°C before shipment on dry ice to the Liverpool Bioanalytical Facility (University of Liverpool, Liverpool, UK). Dolutegravir plasma concentrations were then determined by a validated LC-MS/MS method.^16^ Assays were fully validated in accordance with the FDA Bioanalytical Method Validation, Guidelines for Industry (2018). Inter- and intra-assay accuracy and precision were <10% for all quality control levels. The lower limit of quantification (LLQ) was 10 ng/mL for the Pittsburgh and DolACT studies and 0.75 ng/mL for SSAT061.

### Pharmacokinetic modelling

Nonlinear mixed effects modelling was applied (NONMEM v.7.5.1, ICON PLC, Dublin, Republic of Ireland) to dolutegravir plasma concentration-time data (dolutegravir alone for the DolACT study) from the HIV-negative individuals using Laplacian estimation. Dolutegravir concentrations below the assay LLQs were included using the M3 method (estimating the probability of a sample being below the LLQ).^17–19^ The modelling process was conducted and documented with Pirana (v.2.9.0) interfaced with NONMEM and Perl-speaks-NONMEM (PsN; v.3.4.2), RStudio (v.2023.06.02) and Xpose4^20,21^ used to aid data visualization and model evaluation.

A previously published dolutegravir model,^22^ also developed in volunteers living without HIV, was used as a point of reference and adapted to our data, more specifically to incorporate fitting of concentrations following dolutegravir cessation and below assay LLQ (Pittsburgh and SSAT061). The two-compartment model included a different absorption lag time for fasted and fed dolutegravir administration with interindividual variability on apparent oral clearance (CL/F), interoccasion variability on absorption rate constant (*k*_a_), lag and relative bioavailability (with the pre-dose being treated as a separate occasion) and a combined proportional-additive residual error, with the additive component restricted to 20% of the assay LLQ. Covariate effects included weight via allometric scaling, rifampicin on CL/F (increased with rifampicin) and sex on *k*_a_ (reduced in males). The overall two-compartment model structure was retained for the current analysis and the estimate of fed lag time used as the initial lag parameter (as drug intake was under fed conditions) for re-estimation with bodyweight included on clearance and distribution parameters using allometric scaling *a priori*. Interindividual and interoccasion variability (IIV/IOV) as outlined by Kawuma *et al.*^22^ in addition to simplification of random effects was evaluated. The impact of sex (female versus male) on model parameters was also assessed.

### Simulations

The model was used to simulate dolutegravir pharmacokinetic profiles at a standard recommended dose of 50 mg once daily (7 doses) and for four dose reduction strategies in 1000 individuals of 50, 70 and 90 kg. The alternative doses explored included half dose (25 mg) once daily (seven doses), standard dose (50 mg) every other day (seven doses), standard dose (50 mg) for 4 days on therapy followed by 3 days off (4:3; eight doses) and standard dose (50 mg) for 5 days on therapy followed by 2 days off (5:2; 10 doses). The proportions of trough concentrations (24 hours after dose 7 for standard and half-dose dolutegravir; 48 hours after dose 7 for every other day dosing; 96 hours after dose 8 for 4:3 dosing and 72 hours after dose 10 for 5:2 dosing) above the EC_90_ of 320 ng/mL^23^ were determined and compared along with predicted time below the EC_90_ threshold before the next due dose, to provide a guide of the potential pharmacokinetic robustness of the dose reduction approaches.

## Results

### Analysis population

In total, 73 individuals were included providing 825 dolutegravir concentrations. Overall, 60.3% were female (*n* = 44), just under half were black (49.3%) with age and weight ranging between 20 and 67 years and 41.5 and 97.5 kg, respectively. Demographics stratified by study are summarized in Table 1.

**Table 1.** Summary of demographics of healthy volunteers included in the dolutegravir population pharmacokinetic model [median (range) unless stated otherwise]

Parameter	Pittsburgh	SSAT061	DolACT	Combined
*n*	30	17	26	73
Sex at birth [*n* (%)]				
Male	8 (26.7)	5 (29.4)	16 (61.5)	29 (39.7)
Female	22 (73.3)	12 (70.6)	10 (38.5)	44 (60.3)
Age (years)	37.5 (20.0–67.0)	38.8 (25.9–52.5)	29.0 (20.0–38.0)	33.3 (20.0–67.0)
Weight (kg)	72.1 (49.0–97.5)	74.2 (50.8–97.0)	59.0 (41.5–76.5)	64.4 (41.5–97.5)
Ethnicity [*n* (%)]				
White	26 (86.7)	8 (47.1)	—	34 (46.6)
Black	1 (3.3)	9 (52.9)	26 (100)	36 (49.3)
Asian	3 (10.0)	—	—	3 (4.1)

For the Pittsburgh study, one individual withdrew on day 2 but the collected pharmacokinetic data (day 1 and 2 pre-dose samples) were included. One sample was excluded due to detectable dolutegravir for day 1 pre-dose; all the remaining pre-dose samples on day 1 were below the assay LLQ (<10 ng/mL). Of the remaining concentrations, *n* = 1 at 72 and 96 hours, *n* = 26 at 168 hours and *n* = 29 at 336 hours following the final dose were below the LLQ. For SSAT061 *n* = 3 samples were below the assay LLQ (<0.75 ng/mL), *n* = 1 at 168, 192 and 216 hours post-drug cessation within the same individual. For DolACT, a 4 hour and a 6 hour sample were missing for two separate individuals and therefore excluded. All the remaining dolutegravir concentrations were quantifiable.

### Pharmacokinetic modelling

In line with the two-compartment model structure (Figure S1, available as Supplementary data at *JAC* Online), a combined error model was retained. Duplication of the random effects to include IIV on CL/F and IOV on *k*_a_, absorption lag time and relative bioavailability produced high shrinkage (>30%) for IOV *k*_a_ and absorption lag. Various combinations of random effects were investigated with IIV on CL/F and *k*_a_ and IOV on relative bioavailability producing the most parsimonious model with lower shrinkage (between 8.8% and 27%) and adequate fit to the data. Males had a 72.6% lower *k*_a_ than females (typical values 0.71 versus 2.58 h^−1^). Final model parameters are summarized (Table 2) and prediction-corrected visual predictive check (pcVPC) shown (Figure S2).

**Table 2.** Estimates and relative standard error of the final dolutegravir model

Parameter	Estimate	95% CI^a^
Fixed effects		
CL/F (L/h)	0.787	0.751–0.824
*V*_c_/F (L)	15.0	14.2–15.2
Q/F (L/h)	0.0093	0.0072–0.011
*V*_p_/F (L)	0.671	0.586–0.756
*k*_a_ (h^−1^)	2.58	0.246–4.92
Lag time (h)	0.836	0.776–0.897
Relative bioavailability (F)	1 fixed	—
Covariates		
Weight on CL/F & Q/F^b^	0.75 fixed	—
Weight on *V*_c_/F & *V*_p_/F^b^	1 fixed	—
Male sex on *k*_a_	0.274	0.122–0.426
Random effects		
IIV CL/F (%)	21.5	17.6–24.8
IIV *k*_a_ (%)	136	111–157
IOV F (%)	30.3	24.4–35.2
Residual variability		
Proportional (%)	22.3	19.8–24.7
Additive (mg/L)	0.0013	0.00058–0.0019

^a^95% Bootstrap with replacement (*n* = 1000).

^b^Allometric scaling.

*V*_c_/F, *V*_p_/F, apparent volume of distribution of the central and peripheral compartments; Q/F, intercompartmental clearance; F, relative bioavailability

### Simulations

**Figure 1.**
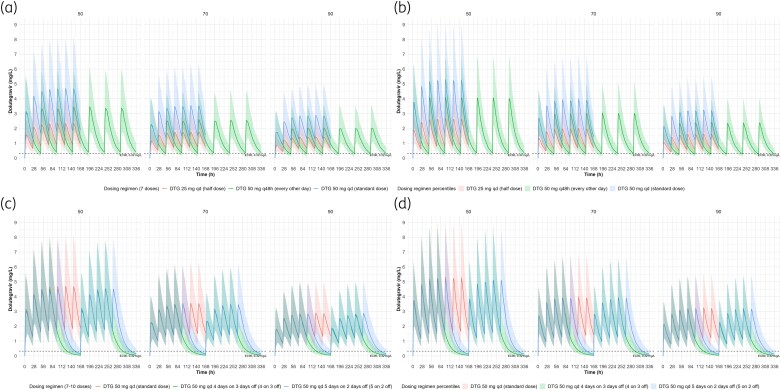
Simulated dolutegravir for standard dose, half dose, every other day in (a) males and (b) females and standard dose with 4 days on 3 days off and 5 days on 2 days off in (c) males and (d) females stratified by weight.

**Figure 2.**
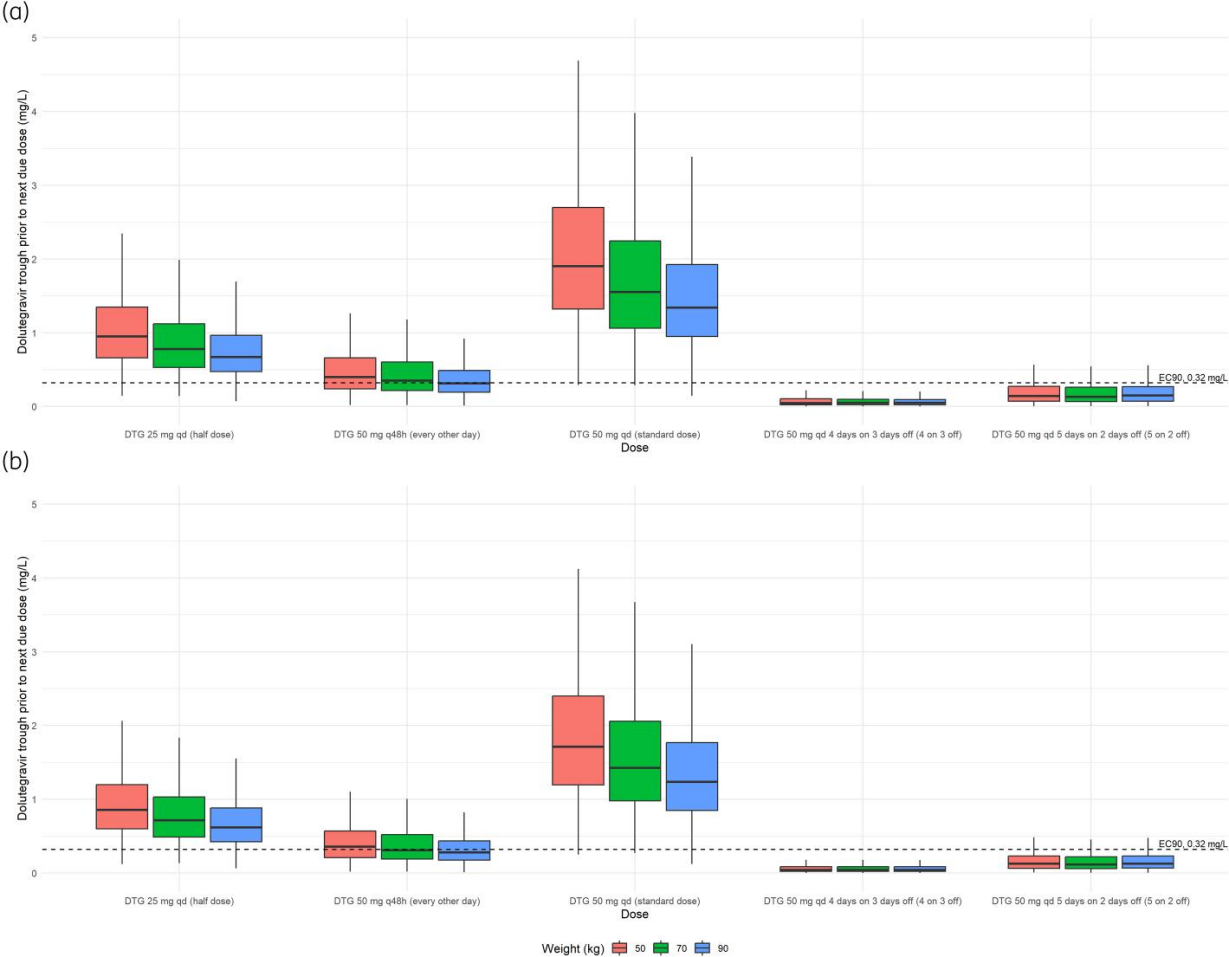
Simulated dolutegravir trough concentrations before the next due dose per dosing regimen stratified by weight in (a) males and (b) females.

Simulations of the reduced-dose and standard dolutegravir in males and females, stratified by weight are illustrated (Figure 1). Simulated trough concentrations before the next due dose (24 or 48 hours after final simulated dose for daily and every other day dosing, respectively, and 96 and 72 hours after final simulated 4:3 and 5:2 dosing, respectively) are also shown (Figure 2). Of the simulated trough concentrations, >88.7% of simulated troughs were above the EC_90_ threshold (>320 ng/mL; Table 3) for half a TLD tablet (25 mg of dolutegravir once daily), whereas 50 mg every other day achieved adequate trough concentrations in 41.6%–62.1% of the simulated populations (Table 3). Markedly lower proportions of simulated trough concentrations above the EC_90_ before the next due dose were observed for 4:3 and 5:2 regimens between 2.0% and 5.2% and 14.1% and 20.7%, respectively (Table 3). For completion and by way of comparison, the model by Kawuma *et al.* was implemented (fed absorption lag, male *k*_a_, no rifampicin) and simulated profiles, troughs and proportions of trough above the EC_90_ shown (Table S1, Figures S3 and S4). Proportions of simulated troughs above the EC_90_ were between 69–80% for 25 mg once daily, 23%–32% for 50 mg every other day and ∼1% and between 5% and 7% for 50 mg dosed 4:3 and 5:2, respectively (Table S1).

**Table 3.** Proportion of simulated dolutegravir trough concentrations before the next due dose above the EC_90_ threshold (320 ng/mL) for standard dose 50 mg once daily and each of the dose reduction strategies (half dose, every other day, 4 days on 3 days off, 5 days on 2 days off) by weight in males and females. Median (range) predicted times below the EC_90_ threshold are also presented for reduced dolutegravir regimens

		Males			
Weight (kg)	Dosing regimen	*n* above EC_90_	*n* below EC_90_	Proportion above EC_90_ (%)	Time below EC_90_ (h)
50	DTG 50 mg q24h	998	2	99.8	
70	DTG 50 mg q24h	997	3	99.7	
90	DTG 50 mg q24h	991	9	99.1	
50	DTG 25 mg q24h	973	27	97.3	1.99 (0.07–6.42)
70	DTG 25 mg q24h	949	51	94.9	2.61 (0.03–8.78)
90	DTG 25 mg q24h	911	89	91.1	2.45 (0.05–18.68)
50	DTG 50 mg q48h	621	379	62.1	6.92 (0.03–23.95)
70	DTG 50 mg q48h	557	443	55.7	7.11 (0.01–27.96)
90	DTG 50 mg q48h	489	511	48.9	8.58 (0.04–35.45)
50	DTG 50 mg q24h 4 days on 3 days off (4:3)	52	948	5.2	40.31 (0.01–70.90)
70	DTG 50 mg q24h 4 days on 3 days off (4:3)	28	972	2.8	41.69 (1.55–78.12)
90	DTG 50 mg q24h 4 days on 3 days off (4:3)	32	968	3.2	42.63 (0.75–77.22)
50	DTG 50 mg q24h 5 days on 2 days off (5:2)	207	793	20.7	19.56 (0.22–49.61)
70	DTG 50 mg q24h 5 days on 2 days off (5:2)	183	817	18.3	21.07 (0.01–51.79)
90	DTG 50 mg q24h 5 days on 2 days off (5:2)	185	815	18.5	20.26 (0.10–49.58)

		Females			
Weight (kg)	Dosing regimen	*n* above EC_90_	*n* below EC_90_	Proportion above EC_90_ (%)	
50	DTG 50 mg q24h	998	2	99.8	
70	DTG 50 mg q24h	993	7	99.3	
90	DTG 50 mg q24h	987	13	98.7	
50	DTG 25 mg q24h	956	44	95.6	2.50 (0.16–6.53)
70	DTG 25 mg q24h	931	69	93.1	2.57 (0.02–10.43)
90	DTG 25 mg q24h	887	113	88.7	3.35 (0.17–14.61)
50	DTG 50 mg q48h	558	442	55.8	7.35 (0.03–24.32)
70	DTG 50 mg q48h	485	515	48.5	7.74 (0.08–28.26)
90	DTG 50 mg q48h	416	584	41.6	9.64 (0.002–36.29)
50	DTG 50 mg q24h 4 days on 3 days off (4:3)	25	975	2.5	42.29 (1.14–73–72)
70	DTG 50 mg q24h 4 days on 3 days off (4:3)	15	985	1.5	43.43 (1.89–78.31)
90	DTG 50 mg q24h 4 days on 3 days off (4:3)	20	980	2.0	45.14 (0.33–77.68)
50	DTG 50 mg q24h 5 days on 2 days off (5:2)	153	847	15.3	20.84 (0.19–49.65)
70	DTG 50 mg q24h 5 days on 2 days off (5:2)	141	859	14.1	22.29 (0.02–52.87)
90	DTG 50 mg q24h 5 days on 2 days off (5:2)	146	854	14.6	22.11 (0.01–50.26)

DTG, dolutegravir; q24d, once daily; q48h, every 48 hours.

Time spent below the dolutegravir EC_90_ of 320 ng/mL for each reduced dosing strategy are summarized (Table 3). Of the reduced-dose regimens, 4:3 and 5:2 simulated dolutegravir concentrations remained below the EC_90_ for the longest period before the next due dose with medians across weight bands ranging 40.3–42.6 hours (males) and 42.3–45.1 hours (females) for 4:3 and 19.6–21.1 hours (males) and 20.8–22.3 hours (females) for 5:2 (Table 3). Median time below target did not exceed 3.3 and 9.6 hours for daily half dose and 50 mg every other day, respectively (Table 3).

### Conclusions

Dose reduction of daily TLD is not standard of care, and we do not provide evidence that such a strategy is safe and effective. Such evidence can only come from clinical evaluation taken ahead of (or if urgency demands, alongside) any change to current programmatic deployment.

In this study we have modelled (using drug concentration measurements taken from HIV-negative volunteers discontinuing dolutegravir as part of clinical trials) the pharmacokinetic effects of four dose reduction strategies: daily, half-dose TLD, alternate day TLD and TLD given 4 days on, three days off or 5 days on, two days off. The first two of these regimens have never been studied in randomized trials, and 23% of 318 participants in QUATUOR who received intermittent therapy (where successful virological suppression was demonstrated with 4:3 dosing) were on dolutegravir-containing regimens.^4^ The 5:2 strategy has also provided sustained virological suppression, typically with nonnucleoside-based treatment (e.g. efavirenz), although BREATHER-Plus reported inferiority of dolutegravir-based therapy in adolescents (primary endpoint of confirmed viral rebound by week 96). One main difference in BREATHER-Plus study design was the less frequent viral load monitoring reflective of local guidelines compared with at least every 12 weeks in other studies.^5^

When comparing dose reduction strategies, two different adverse outcomes can be considered: viral rebound without resistance (e.g. many patients on TLD will resuppress on this drug after rebound resulting from poor adherence^24^), versus virological failure with the acquisition of resistance mutations. The likelihood of the latter depends on the genetic barrier to resistance of the overall regimen, but also the amount of time spent in the ‘zone of selective pressure’ for resistance: a putative window above which viral replication is completely suppressed, below which virus replicates without advantage from mutations and within which drug selection pressure is applied, preferentially amplifying resistance mutations.

For most antiretrovirals, the zone of selective pressure for resistance is not known. In the case of lamivudine and tenofovir, long intracellular half-lives of their active phosphorylated metabolites will probably confer additional protection. In the SPRING-1 Phase IIb dose-ranging study of dolutegravir, concentration-effect modelling predicted that antiviral responses were blunted at concentrations ∼320 ng/mL.^23^ It is therefore plausible that the upper bound of the zone of selective pressure lies close to this value, i.e. allowing viral replication in the presence of drug.

Our model predicts that average plasma dolutegravir trough concentrations remain well above 320 ng/mL with full- and half-dose TLD administered daily regardless of body weight. a half dose resulted in simulated concentrations below the 320 ng/mL a median of no more than 3.3 hours (but with a maximum of ∼19 hours for 90 kg males). With 1 TLD pill every other day, average trough plasma concentrations approach, or fall below 320 ng/mL at all body weights modelled, with lowest plasma concentrations in 90 kg individuals, however, median time below the target threshold did not exceed 10 hours (maximum ∼36 hours). In the case of a 4:3 or 5:2 regimen, treatment interruption is associated with a steady decline in concentrations [e.g. median of 47 ng/mL (P10-P90: 11–183) at the end of 3 days off dolutegravir in a 70 kg male for 4:3] resulting in median time below the EC_90_ approaching ∼2 days for 4:3 and 1 day for 5:2. These data suggest that the dose reduction regimens examined may not be equivalent in risk of developing resistance, should the viral load rebound. The number of times the target threshold is crossed together with time spent within the zone of selective pressure would need to be considered. In 1 month of treatment, alternate day dosing is associated with mean plasma dolutegravir concentrations that cross the zone of selective pressure for resistance 14 times, 4:3/5:2 regimens cross this zone four times whereas daily treatment (one or half TLD pill) remains above this threshold (potentially even with inaccurate splitting to half-pills). The cumulative length of time over 1 month of treatment would be similar for alternate day dosing and 5:2 (∼100 versus 84 hours) but lengthier for 4:3 (∼167 hours, based on median time below the EC_90_ for a 70-kg male).

This analysis has caveats. The drug concentrations which we used derive from volunteers living without HIV, since pharmacokinetic characterization of dolutegravir washout cannot be easily or ethically studied in people living with HIV. If viral loads do not rebound with intermittent therapy, then mutations will not be selected. The absence of any defined limits makes windows for drug selective pressure an abstract concept that requires proof of clinical relevance. TLD is a regimen with a high genetic barrier to resistance.^25^ Across Africa, TLD is used first-line, as well as second-line (following failure of efavirenz-based therapy) where the risk of rebound with resistance is not the same. However, our model shows these different strategies cannot be assumed to be equally safe or robust and should be separately tested taking into consideration all available evidence for their use, as well as for pragmatic deployment, and the importance of monitoring for treatment failure.

## Supplementary Material

dkaf445_Supplementary_Data

## Supplementary data

Figures S1 to S4 and Table S1 are available as Supplementary data at *JAC* Online.
