# Supplementary material for: A model-based comparison of dose reduction strategies for fixed-dose dolutegravir-containing regimens
Source: J Antimicrob Chemother. 2025 Dec 12;81(1):dkaf445. doi: 10.1093/jac/dkaf445 (PMC12802907; doi:10.1093/jac/dkaf445)
Supplement: dkaf445_Supplementary_Data [file dkaf445_supplementary_data.docx]

**A Model-based Comparison of Dose Reduction Strategies for Fixed-Dose Dolutegravir-containing Regimens**

Supplementary Materials

**Table S1**

Proportion of simulated dolutegravir trough concentrations prior to the next due dose above the EC_90_ threshold (320 ng/mL) using the model by Kawuma et al., (fed absorption lag, male volunteers, in the absence of rifampicin) [1] for standard dose 50 mg once daily and each of the dose reduction strategies (half dose, every other day, 4 days on 3 days off, 5 days on 2 days off) by weight.

| **Weight (kg)** | **Dosing regimen** | **N above EC_90_** | **N below EC_90_** | **Proportion above EC_90_ (%)** |
| --- | --- | --- | --- | --- |
| 50 | DTG 50 mg q24h | 968 | 32 | 96.8 |
| 70 | DTG 50 mg q24h | 950 | 50 | 95 |
| 90 | DTG 50 mg q24h | 930 | 70 | 93 |
| 50 | DTG 25 mg q24h | 797 | 203 | 79.7 |
| 70 | DTG 25 mg q24h | 740 | 260 | 74 |
| 90 | DTG 25 mg q24h | 685 | 315 | 68.5 |
| 50 | DTG 50 mg q48h | 315 | 685 | 31.5 |
| 70 | DTG 50 mg q48h | 272 | 728 | 27.2 |
| 90 | DTG 50 mg q48h | 227 | 773 | 22.7 |
| 50 | DTG 50 mg q24h 4 days on 3 days off (4:3) | 12 | 988 | 1.2 |
| 70 | DTG 50 mg q24h 4 days on 3 days off (4:3) | 6 | 994 | 0.6 |
| 90 | DTG 50 mg q24h 4 days on 3 days off (4:3) | 5 | 995 | 0.5 |
| 50 | DTG 50 mg q24h 5 days on 2 days off (5:2) | 66 | 934 | 6.6 |
| 70 | DTG 50 mg q24h 5 days on 2 days off (5:2) | 51 | 949 | 5.1 |
| 90 | DTG 50 mg q24h 5 days on 2 days off (5:2) | 57 | 943 | 5.7 |

DTG: dolutegravir; q12d: once daily; q48h: every 48 hours

**Figure S1**

Schematic of the dolutegravir 2-compartment oral model

**k_23_**

**Q/F**

**GUT**

**CENTRAL**

**V_c_/F**

**CL/F**

**k_a_**

**ALAG1**

**PERIPHERAL**

**V_p_/F**

**k_32_**

k_a_: absorption rate constant; ALAG1: absorption lag-time; CL/F: apparent oral clearance; V_c_/F: apparent volume of distribution of the central compartment; Q/F: apparent intercomparmental clearance; V_p_/F: apparent volume of the peripheral compartment; k_23_: transfer rate constant from the central to peripheral compartment; k_32_: transfer rate constant from the peripheral to central compartment

**Figure S2**

Dolutegravir prediction-corrected visual predictive check (pcVPC) for (**A**) Pittsburgh study (n=30), (**B**) SSAT061 (n=17) and (**C**) DolACT (n=26), also with the proportion below the lower limit of quantification (BLQ; for Pittsburgh and SSAT061). Pittsburgh and SSAT061 were following dolutegravir cessation. The lines represent the percentiles of the observed data (P5, P50, P95) and the shaded areas the 95% CI of the simulated data. Observed concentration-time data for the Pittsburgh study (350 concentrations), SSAT061 (272 concentrations) and DolACT (208 concentrations) are superimposed (open circles).

| 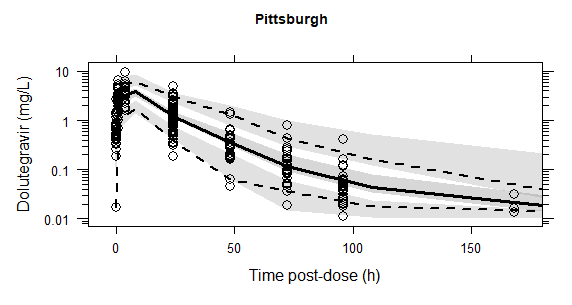  **A** | 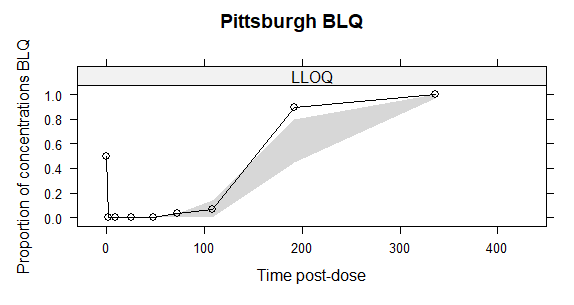 |
| --- | --- |
| 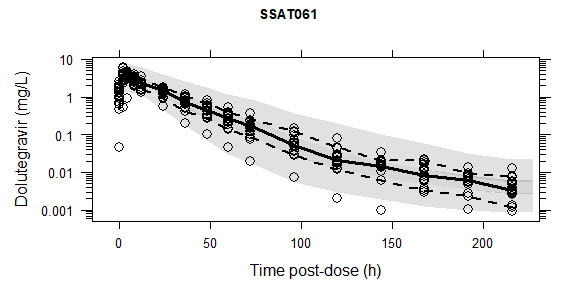  **B** | 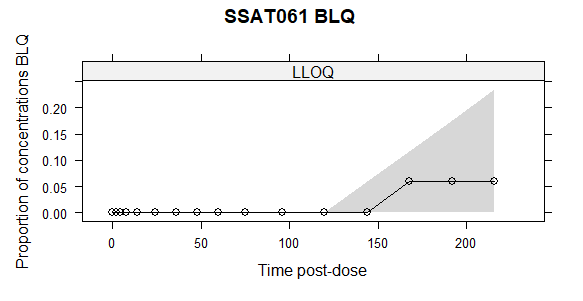 |
| 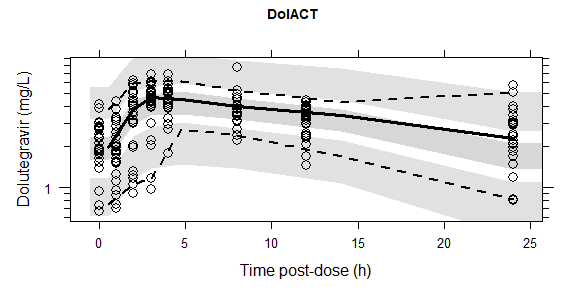  **C** |  |

**Figure S3**

Simulated dolutegravir (DTG) for (A) standard dose (50 mg once daily), half dose (25 mg once daily), every other day (50 mg every 48 hours) and (B) standard dose (50 mg once daily) with 4 days on 3 days off (50 mg for 4 days, 3 days off treatment) and 5 days on 3 days off (50 mg for 5 days, 2 days off treatment) stratified by weight (50, 70 and 90 kg) implementing the model by Kawuma *et al.*, (*Antimicrob Agents Chemother* 2019; 66 (**6**): e0021522), under fed conditions, male absorption rate constant, in the absence of rifampicin.


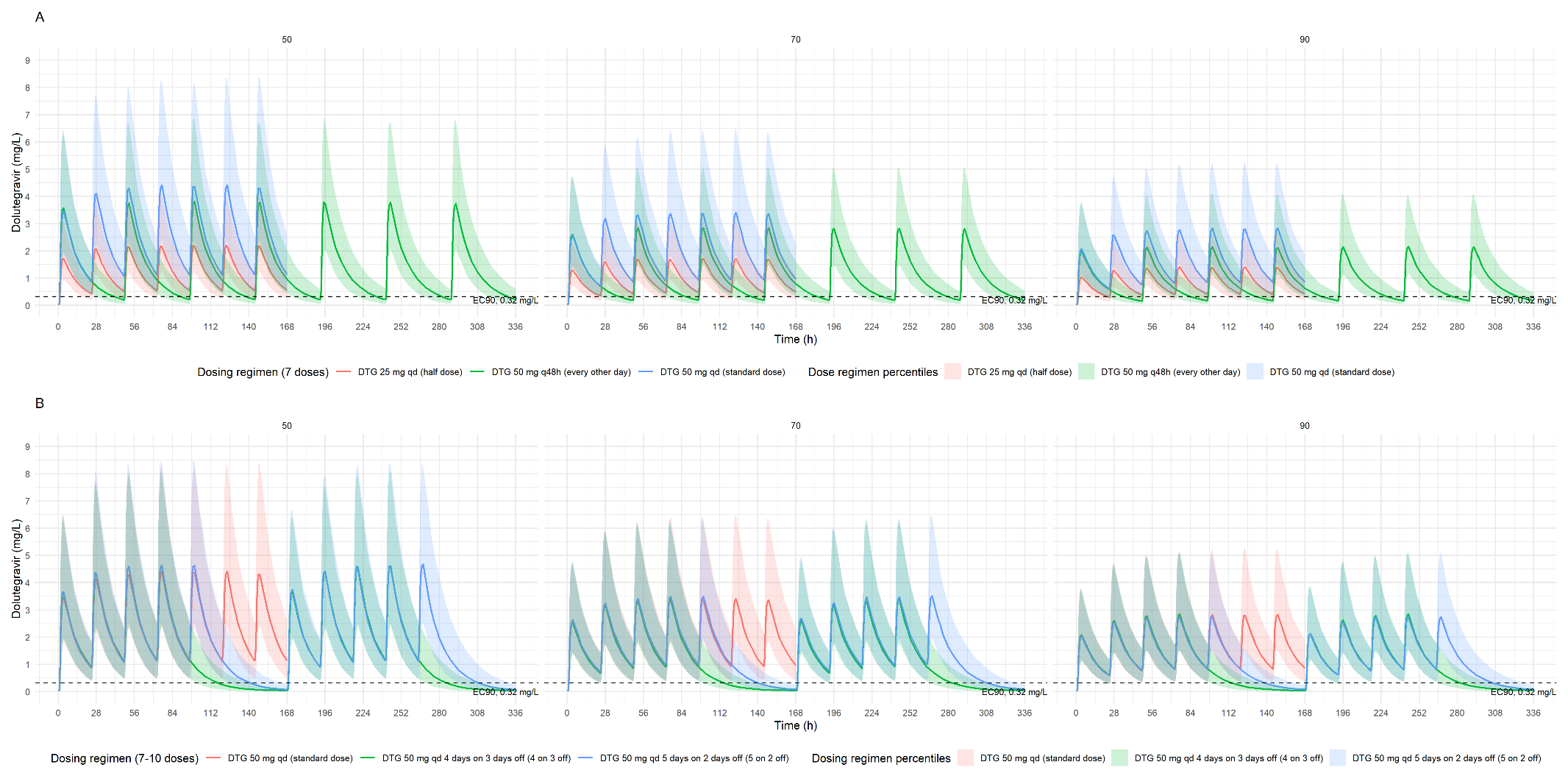


**Figure S4**

Simulated dolutegravir (DTG) trough concentrations prior to the next due dose per dosing regimen stratified by weight (50, 70 and 90 kg) implementing the model by Kawuma *et al.*, (*Antimicrob Agents Chemother* 2019; 66 (**6**): e0021522), under fed conditions, male absorption rate constant, in the absence of rifampicin.


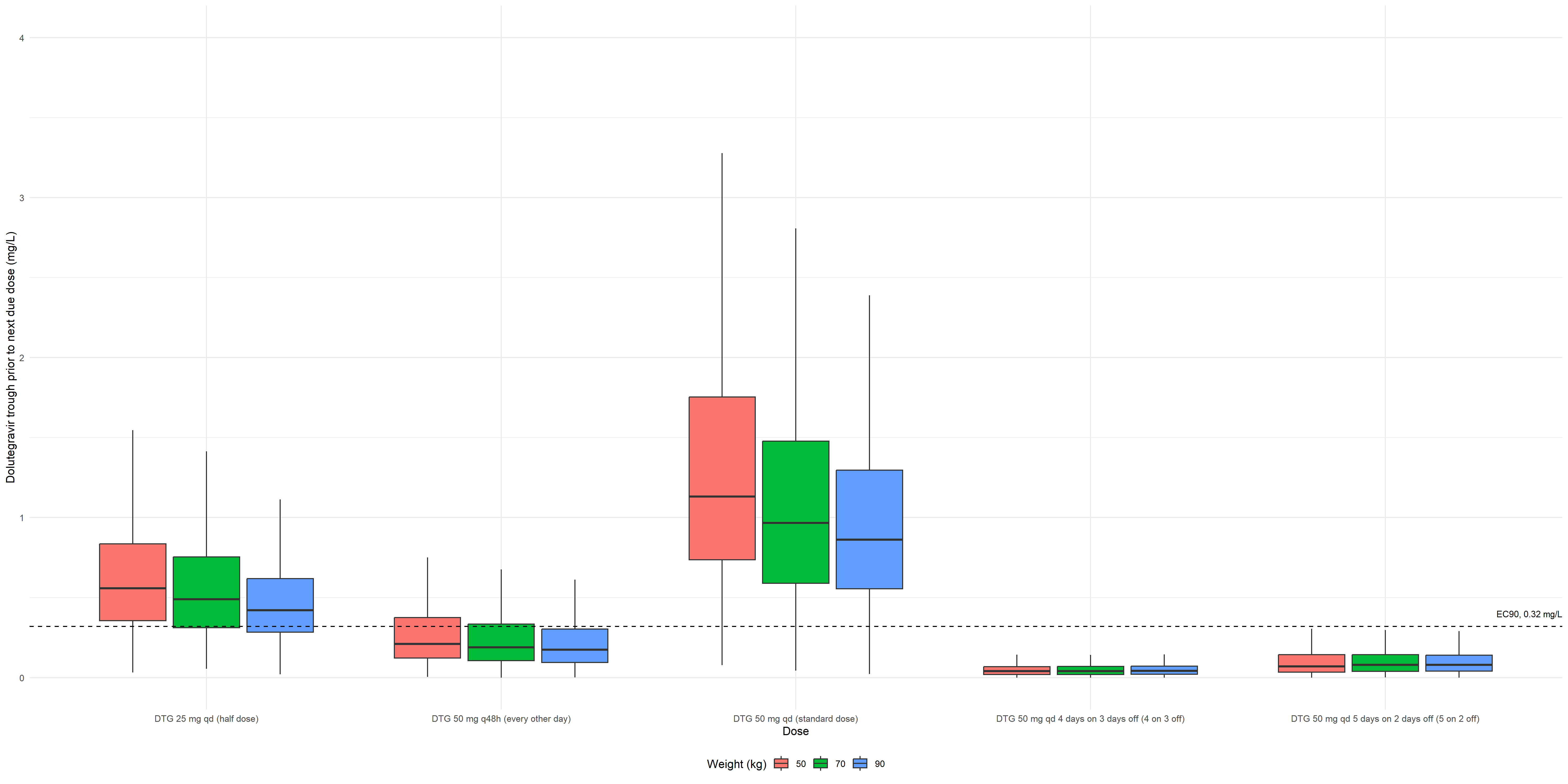


**References**

1. Kawuma AN, Wasmann RE, Dooley KE, Boffito M, Maartens G, Denti P. Population Pharmacokinetic Model and Alternative Dosing Regimens for Dolutegravir Coadministered with Rifampicin. Antimicrob Agents Chemother. 2022; **66**: e0021522.
